# Introduction and Spread of Dengue Virus 3, Florida, USA, May 2022–April 2023

**DOI:** 10.3201/eid3002.231615

**Published:** 2024-02

**Authors:** Forrest K. Jones, Andrea M. Morrison, Gilberto A. Santiago, Kristyna Rysava, Rebecca A. Zimler, Lea A. Heberlein, Edgar Kopp, Florida Department of Health Bureau of Public Health Laboratory Team,, Katharine E. Saunders, Samantha Baudin, Edhelene Rico, Álvaro Mejía-Echeverri, Emma Taylor-Salmon, Verity Hill, Mallery I. Breban, Chantal B.F. Vogels, Nathan D. Grubaugh, Lauren M. Paul, Scott F. Michael, Michael A. Johansson, Laura E. Adams, Jorge Munoz-Jordan, Gabriela Paz-Bailey, Danielle R. Stanek

**Affiliations:** Centers for Disease Control and Prevention, San Juan, Puerto Rico, USA (F.K. Jones, G.A. Santiago, M.A. Johansson, L.E. Adams, J. Munoz-Jordan, G. Paz-Bailey, K. Rysava);; Centers for Disease Control and Prevention, Atlanta, Georgia, USA (F.K. Jones, K.E. Saunders);; Florida Department of Health, Tallahassee, Florida, USA (A.M. Morrison, R.A. Zimler, L.A. Heberlein, E. Kopp, K.E. Saunders, S. Baudin, E. Rico, Á. Mejía-Echeverri, D.R. Stanek);; Yale School of Medicine, New Haven, Connecticut, USA (E. Taylor-Salmon);; Yale School of Public Health, New Haven (E. Taylor-Salmon, V. Hill, M.I. Breban, C.B.F. Vogels, N.D. Grubaugh);; Florida Gulf Coast University, Fort Myers, Florida, USA (L.M. Paul, S.F. Michael)

**Keywords:** dengue virus, DENV, DENV-3, vector-borne infections, viruses, whole-genome sequencing, phylogenetics, transmission reconstruction, Florida, United States

## Abstract

During May 2022–April 2023, dengue virus serotype 3 was identified among 601 travel-associated and 61 locally acquired dengue cases in Florida, USA. All 203 sequenced genomes belonged to the same genotype III lineage and revealed potential transmission chains in which most locally acquired cases occurred shortly after introduction, with little sustained transmission.

Dengue virus (DENV) is not endemic in the continental United States (*1*); most cases occur among travelers to DENV-endemic areas (*2*). In Florida, USA, DENV infections are primarily reported among travelers (https://ndc.services.cdc.gov/case-definitions/dengue-virus-infections-2015); however, locally acquired cases and limited outbreaks have been reported in Monroe County in 2009–2010 (n = 88), Martin County in 2013 (n = 24), and Monroe County in 2020 (n = 72) (*3*–*5*). During 2009–2021, an annual median of 83 (range 19–413) travel-associated DENV infections and 7 (range 0–77) locally acquired cases were reported in Florida; all DENV types (DENV-1−4) occurred among both travel-associated and locally acquired cases (*6*). Previous work demonstrated the DENV vectors *Aedes aegypti* and *A. albopictus* mosquitoes are present across Florida (*7*).

In early 2022, the Florida Department of Health (FDOH) identified an increase in travel-associated DENV infections, primarily among travelers returning from Cuba. In July 2022, a DENV-3 outbreak was reported in Cuba (*8*); DENV-3 case increases were also documented in other countries in the Americas (*9*,*10*). On July 18, Miami-Dade County health officials issued a mosquito-borne illness advisory after the first locally acquired DENV infection in 2022 was confirmed in a Florida resident (*11*). We document the DENV-3 outbreak in Florida by describing the epidemiologic features of reported cases, analyzing DENV-3 genomic sequences, and reconstructing possible transmission trees.

## The Study

FDOH routinely conducts active case-finding activities for DENV and conducts IgM and reverse transcription PCR testing for confirmation and DENV serotype identification. Suspected case-patients are interviewed to identify risk factors, possible mosquito exposure locations, and additional suspected cases (*3*). Ethics approval was not required because this work was part of standard public health outbreak surveillance and response. 

**Figure 1:**
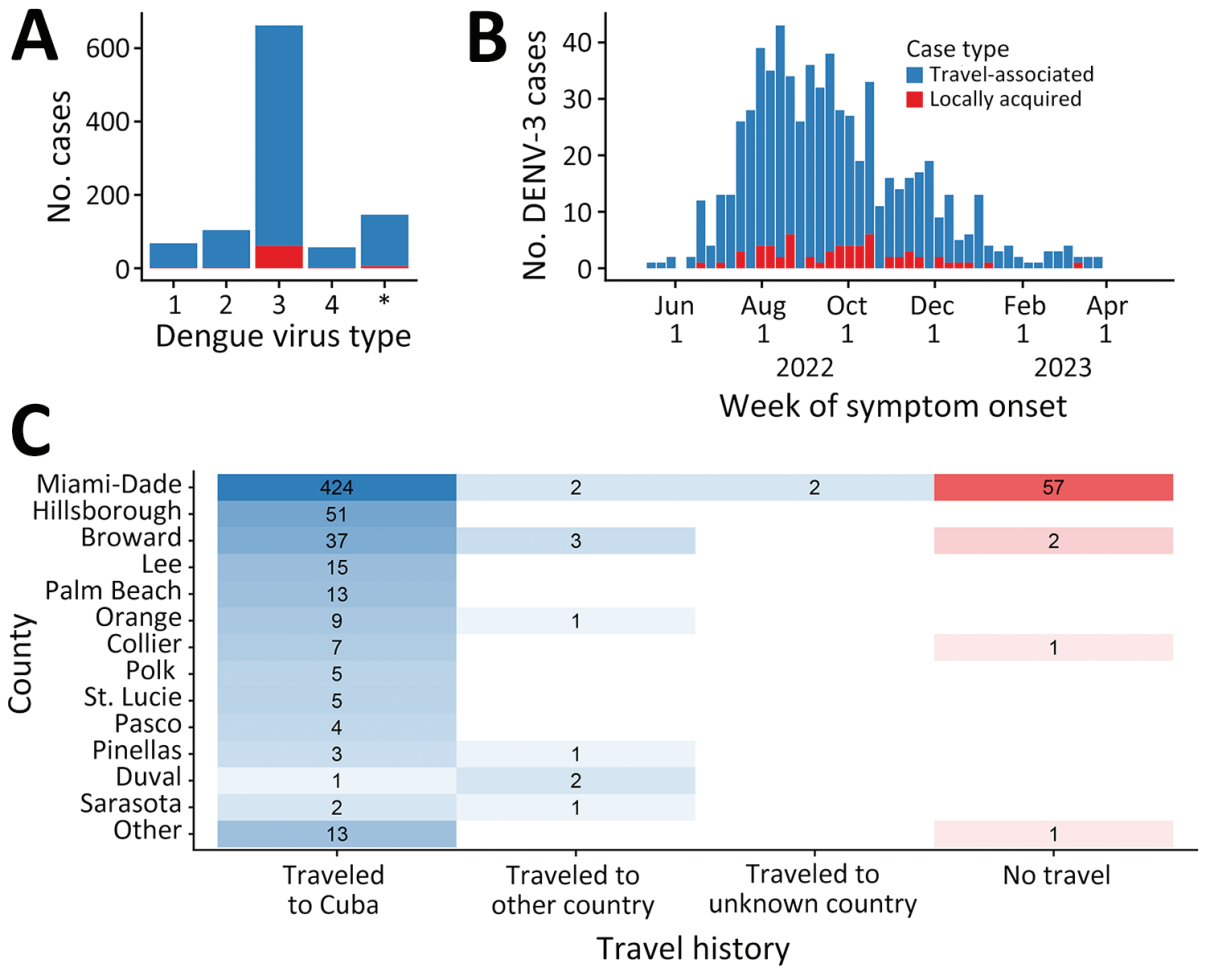
DENV serotype distribution and DENV-3 case distribution by week of symptom onset, county of reporting, and origin of travel, Florida, USA, May 1, 2022–April 30, 2023. A) Number of dengue cases by each virus serotype. Cases with an unknown dengue virus type (asterisk) only had a positive serologic test or multiple serotypes identified. B) Epidemic curve of reported cases of DENV-3, showing 601 travel-associated cases and 61 locally acquired cases. C) Heat map indicating number of DENV-3 cases by county and by travel history. Other countries were Bangladesh, Colombia, Guyana, India, Jamaica, Mexico, Pakistan, and Sri Lanka. The names of counties reporting >3 DENV-3 cases are shown and sorted by the total number of cases reported. DENV, dengue virus; DENV-3, DENV serotype 3.

During May 1, 2022–April 30, 2023 (52 weeks), 1,037 DENV infections were reported, 966 (93%) were travel-associated and 71 (7%) locally acquired. DENV-3 was the most frequently identified serotype (64%, n = 662), followed by DENV-2 (10%, n = 104), DENV-1 (7%, n = 68), and DENV-4 (5%, n = 57); in 146 (14%) cases, multiple serotypes or no serotype was identified (Figure 1, panel A). Among DENV-3 cases, 601 (91%) were travel-associated and 61 (9%) were locally acquired cases (Figure 1, panel B). Most DENV-3 case-patients identified as White (n = 609; 92%) and Hispanic or Latino (n = 642, 97%).

Among 601 travel-associated DENV-3 cases, the median age was 52 (interquartile range 41–61) years; 51% of patients were male and 49% female. Most (98%, n = 589) case-patients with travel-associated DENV-3 had recently traveled from Cuba; they were reported in 21/67 Florida counties (Figure 1, panel C). Miami-Dade County had the most travel-associated DENV-3 cases (71%, n = 428). Among 61 locally acquired DENV-3 cases, the median age was 54 (interquartile range 36–58) years; 67% of patients were male and 33% female, and nearly all (93%, n = 57) were reported in Miami-Dade County. The 485 DENV-3 case-patients in Miami-Dade County were identified in 60/82 postal (ZIP) codes.

We performed genomic characterization of DENV-3 by sequencing the complete genomes of 203 cases at the Centers for Disease Control and Prevention (San Juan, Puerto Rico, USA), Yale School of Public Health (New Haven, CT, USA), and FDOH (Appendix 1) (*12*). Sequencing was prioritized and successful for 34 locally acquired cases, as well as case-patients with recent travel history to Cuba (n = 168) or Guyana (n = 1). To assess the representativeness of DENV sequences, we evaluated symptom onset dates and counties of residence for cases selected for sequencing and all cases detected (Appendix 1 Figure 1). We conducted maximum-likelihood phylogenetic analysis to infer the genetic relatedness of DENV-3 to contemporary circulation globally. Global context was provided with a subsample of 146 publicly available genomes that represent relevant genotypes.

**Figure 2:**
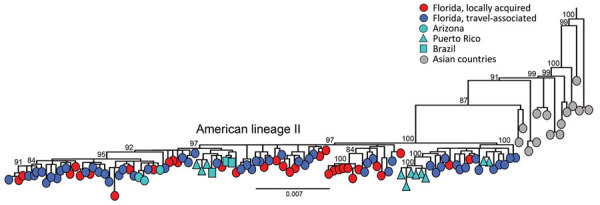
Evolutionary analysis of dengue virus serotype 3 sampled in Florida, USA, May 1, 2022–April 30, 2023. Maximum-likelihood phylogenetic tree was generated from a subset of 203 complete genomes from Florida (34 local cases, 168 cases in persons with recent travel history to Cuba, and 1 traveler case from Guyana) and 146 complete genomes publicly available (1985–2022) from GenBank representing genotype III, American lineage II. A subset of the sequences was used because of the low diversity in the population sample, which was limiting the phylogenetic signal and hampering the statistical analyses that supported the tree accuracy and certainty in major nodes. Sampling locations are coded by shape and color. Scale bar represents nucleotide substitutions per site.

The DENV-3 genomes identified in Florida are classified as genotype III and cluster within the novel American II lineage (*9*). We observed a close relationship with DENV-3 genomes recently identified in Arizona, Puerto Rico, Guyana, and Brazil, indicating that the lineage is spreading across the Americas (Figure 2). However, the limited sampling of the new American II lineage prevented us from inferring a potential time of emergence in Florida. The short branch lengths and similarity between locally acquired and travel-associated cases in the phylogenetic tree demonstrate low genomic diversity during the sampling period, where genomes from locally acquired cases cluster randomly with travel-associated cases. The tree topology suggests frequent importation events occurred during the sampling period and indicate frequent movement of DENV between Cuba and Florida without establishing sustained local transmission in Florida.

To model a possible transmission tree, we adapted a graph-based model using genomic sequences and symptom onset dates from 31 locally acquired and 144 travel-associated cases (Appendix 1) (*13*,*14*). To account for infections in transmission chains that went undetected between reported cases, we included a surveillance reporting probability (i.e., the probability an infection was detected as a case) and performed sensitivity analyses assuming different reporting probabilities of 1%, 5%, 10%, and 15%. Assuming a 5% reporting probability, we identified 22 travel-associated cases (15%) with most compatible linkages leading to the 31 locally acquired cases (Appendix 1 Figure 2). Overall, 122 (85%) travel-associated cases had no likely linkage to locally acquired cases, 17 (11%) were linked to 1 case, 2 (1%) were linked to 2 cases, 2 (1%) were linked to 3 cases, and 1 (1%) was linked to 4 cases.

## Conclusions

We documented an unprecedented number of travel-associated and locally acquired DENV-3 cases in Florida during May 2022–April 2023; circulation of the DENV-3 genotype III was recently identified in the Americas. Our investigation illustrates that local transmission and spread in Florida was limited, despite multiple introductions from outside the country. Sequencing and phylogenetic analysis revealed that cases were from the same DENV-3 genotype III lineage and were highly related to one another and to cases identified in Puerto Rico, Arizona, and Brazil. Assessment of possible linkages between sequenced cases indicated that local transmission during this outbreak was limited; most travel-associated cases did not lead to further transmission.

DENV activity in Cuba and Florida are linked given their proximity and the extensive travel between them. Our results are similar to findings in Florida in 2019 (*5*), where many DENV case-patients reported recent travel to Cuba, leading to an increase in locally acquired cases. An elevated number of locally acquired DENV cases in Florida might be expected after a high number of introductions, but our analysis suggests that DENV introductions did not result in sustained local transmission beyond small-scale outbreaks. Factors potentially reducing transmission include living conditions (e.g., use of air conditioning and screens), rapid case notification that enabled vector interventions (e.g., spraying insecticide, conducting surveillance, community education, and removing standing water), or limited availability of mosquito breeding sites (*15*).

The relatively low genetic diversity in this dataset limited our ability to estimate the timing of initial DENV-3 introductions and fully reconstruct local spread. We did not use case locations to determine the compatibility of transmission links. DENV case detection continued through 2023 in Florida; efforts to understand those transmission dynamics are ongoing.

In summary, we used epidemiologic surveillance and genomic sequencing to identify a newly emerging lineage of DENV-3 genotype III that caused an unusually large number of travel-associated and locally acquired DENV infections in Florida, particularly in Miami-Dade County. Our analysis suggests that locally acquired cases were driven by large numbers of case-patients with recent travel to Cuba and that DENV persistence in Florida was limited. Close monitoring of DENV activity internationally, as well as increasing healthcare provider awareness about DENV identification and testing, can strengthen preparedness and response to future introductions in non–DENV-endemic areas.

Appendix 1 Additional information about introduction and spread of dengue virus 3, Florida, USA, May 2022–April 2023
