# Supplementary material for: Introduction and Spread of Dengue Virus 3, Florida, USA, May 2022–April 2023
Source: Emerg Infect Dis. 2024 Feb;30(2):376–9. doi: 10.3201/eid3002.231615 (PMC10826764; doi:10.3201/eid3002.231615)
Supplement: Appendix 1 — Additional information about introduction and spread of dengue virus 3, Florida, USA, May 2022–April 2023 [file 23-1615-Techapp-s1.pdf]

Article DOI: https://doi.org/10.3201/eid3002.231615

EID cannot ensure accessibility for supplementary materials supplied by authors. Readers who have difficulty accessing supplementary content should contact the authors for assistance.

Introduction and Spread of Dengue Virus 3, Florida, USA, May 2022–April 2023

Appendix 1

Methods

Sequencing methods

Ethics

Sequencing of de-identified remnant clinical specimens that tested positive for dengue virus was done at the Yale School of Public Health. The Institutional Review Boards (IRB) from the Yale University Human Research Protection Program determined that pathogen genomic sequencing of de-identified remnant diagnostic samples as conducted in this study is not research involving human subjects (Yale IRB Protocol ID: 2000033281).

Dengue virus sequencing

At the Yale School of Public Health, dengue virus RNA was sequenced using a pan-serotype, highly multiplexed PCR approach, called ‘DengueSeq’ (a derivative of PrimalSeq) (*1*). A detailed protocol, including the DENV1-4 primer schemes, was previously published (*2*). In brief, we prepared libraries using the Illuminia COVIDSeq test (RUO version) with the DENV1-4 primers, and pooled libraries were sequenced on the Illumina NovaSeq (paired-end 150), targeting 1 million reads per individual library. Consensus genomes were generated at a depth of coverage of 10X and minimum frequency threshold of 0.75 using a custom DengueSeq bioinformatics pipeline (*3*), which includes iVar (*4*). All DENV genomes and sequencing data are available on BioProject PRJNA951702.

At the CDC Dengue Branch, dengue virus RNA was sequenced using dengue virus serotype 3 (DENV-3) specific custom-designed PCR primers. A detailed protocol was previously described (*5*). Briefly, we prepared libraries using the NEBNext Ultra II DNA Library Prep Kit for Illumina with DENV-3 primers, and pooled libraries were sequenced on the Illumina MiSeq (paired-end 600). Consensus genomes were generated using a custom DengueSeq bioinformatics pipeline, which includes iVar.

Transmission linkage compatibility score calculation

Using sequenced locally acquired and travel-associated cases reported with an onset date in 2022, we reconstructed possible transmission chains building upon methods published in Hampson et al., 2009 (*6*) and Cori et al., 2018 (*7*). For each reported case (*i*), we calculated a compatibility score (p_{ij}) for linkage between the case and its potential sources (*j*) defined as all cases with an onset date preceding case *i* as follows:

$$p_{ij} = \frac{S(t_{ij})\,G(d_{ij})}{\max S(x) \max\, G(x)}$$

S and G represent the probability density function of the dengue serial interval (Gamma distributed) and probability mass function of the case genetic distance accounting for the serial interval (Negative binomial distributed), respectively. Here t_{ij} and d_{ij} represent the temporal and genetic distance between cases *i* and *j,* whilst *x* indicates all possible values of each probability function. To account for unobserved transmission between *i* and *j*, we expand the default probability functions by the reporting probability as described in Cori et al.,2018. Geographic location was not accounted for when determining the compatibility score.

For the distributions of S and G, we applied a pruning approach to correct for biologically unlikely links by setting cutoff values. For the estimated serial interval, we removed all potential case connections with onset dates less than five days apart, and for both the temporal and genetic distance distributions under each assumed reporting probability we removed all pairs greater than the upper 95% quantile (Figure S3). The expected daily mutation rate was 1.9×10^{-2} substitutions per day given the mean number of expected substitutions per nucleotide per year (6.98×10^{-4} [95% PI: 5.39×10^{-4} – 8.62×10^{-4}]) and the whole genome size (10,170) based off the molecular analysis done earlier in this work. To infer parameter values for the serial interval, we fitted a Gamma distribution to 1,000 simulated sums of extrinsic and intrinsic incubation periods

of dengue virus (Chan & Johansson, 2012) (*8*) and a mosquito half-life assuming temperatures of 20–30 degrees Celsius (Brady et al., 2013) (*9*).

For travel-associated cases, we did not attempt to identify the source of their infection as we assumed they were infected outside of Florida. Most compatible linkages for locally acquired cases were identified by identifying the linkage with the highest compatibility score.

References

1. Quick J, Grubaugh ND, Pullan ST, Claro IM, Smith AD, Gangavarapu K, et al. Multiplex PCR method for MinION and Illumina sequencing of Zika and other virus genomes directly from clinical samples. Nat Protoc. 2017;12:1261–76. PubMed https://doi.org/10.1038/nprot.2017.066

2. Vogels C. DengueSeq: A pan-serotype whole genome amplicon sequencing protocol for dengue virus v1 [cited 2023 Sep 27]. https://www.protocols.io/view/dengueseq-a-pan-serotype-whole-genome-amplicon-seq-kqdg39xxeg25/v2

3. Lab G. DENV_pipeline [cited 2023 Sep 27]. https://github.com/grubaughlab/DENV_pipeline

4. Grubaugh ND, Gangavarapu K, Quick J, Matteson NL, De Jesus JG, Main BJ, et al. An amplicon-based sequencing framework for accurately measuring intrahost virus diversity using PrimalSeq and iVar. Genome Biol. 2019;20:8. PubMed https://doi.org/10.1186/s13059-018-1618-7

5. Santiago GA, Flores B, González GL, Charriez KN, Huertas LC, Volkman HR, et al. Genomic surveillance of SARS-CoV-2 in Puerto Rico enabled early detection and tracking of variants. Commun Med (Lond) 2022;2:100. **PMID 35968047**

6. Hampson K, Dushoff J, Cleaveland S, Haydon DT, Kaare M, Packer C, et al. Transmission dynamics and prospects for the elimination of canine rabies. PLoS Biol. 2009;7:e53. **PMID 19278295**

7. Cori A, Nouvellet P, Garske T, Bourhy H, Nakouné, Jombart T. A graph-based evidence synthesis approach to detecting outbreak clusters: an application to dog rabies. PLoS Comput Biol. 2018;14:e1006554. **PMID 30557340**

8. Chan M, Johansson MA. The incubation periods of dengue viruses. PloS One. 2012;7:: e50972. **PMID 23226436**

9. Brady OJ, Johansson MA, Guerra CA, Bhatt S, Golding N, Pigott DM, et al. Modelling adult *Aedes aegypti* and *Aedes albopictus* survival at different temperatures in laboratory and field settings. Parasit Vectors. 2013;6:351. PubMed https://doi.org/10.1186/1756-3305-6-351

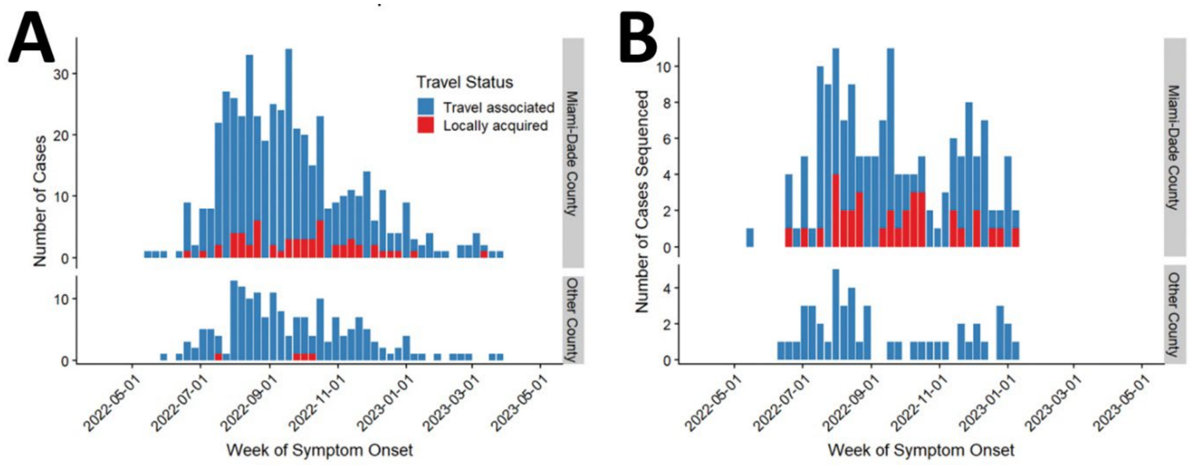

Appendix 1 Figure 1. Epidemic curve of all dengue virus 3 (DENV-3) cases (A) and sequenced cases (B). A) The epidemic curve of all DENV-3 cases is provided, with the top panel showing cases from Miami-Dade County and the bottom panel showing cases from all other counties. B) The subset of these cases which have been sequenced is shown.

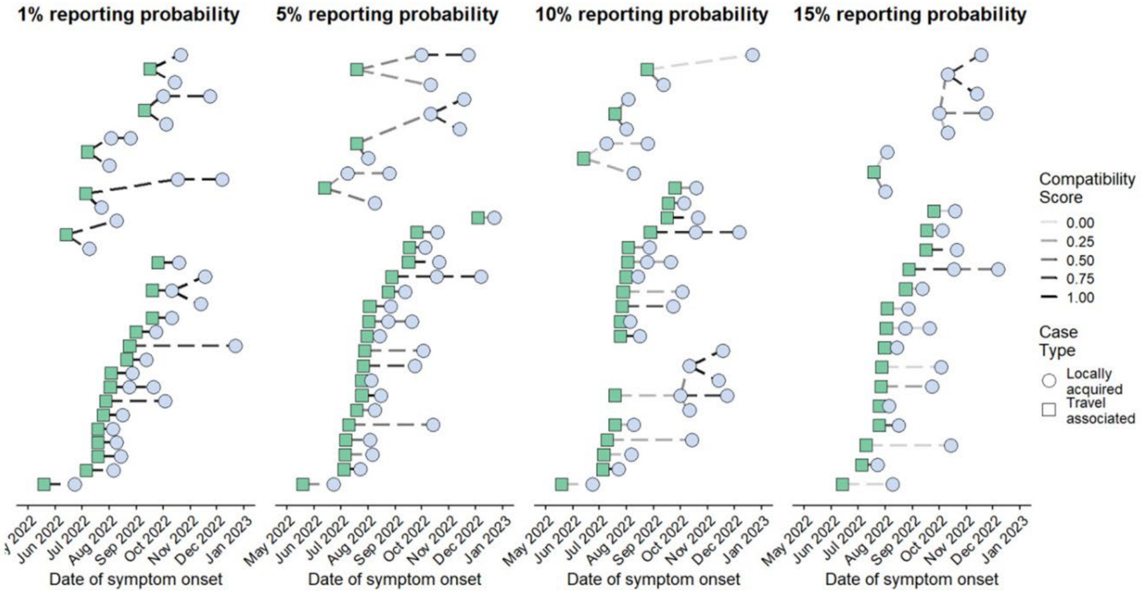

Appendix 1 Figure 2. Reconstructed transmission trees for 1%, 5%, 10%, and 15% reporting probabilities. Undetected infections may have occurred in between linkages (as indicated by the dashed lines). Compatibility scores (as calculated in Methods) ranged from 0 to 1, with darker dashed lines indicating higher compatibility. Only maximum compatibility linkages are shown on the graph. Cases without linkages are not represented on the graph.

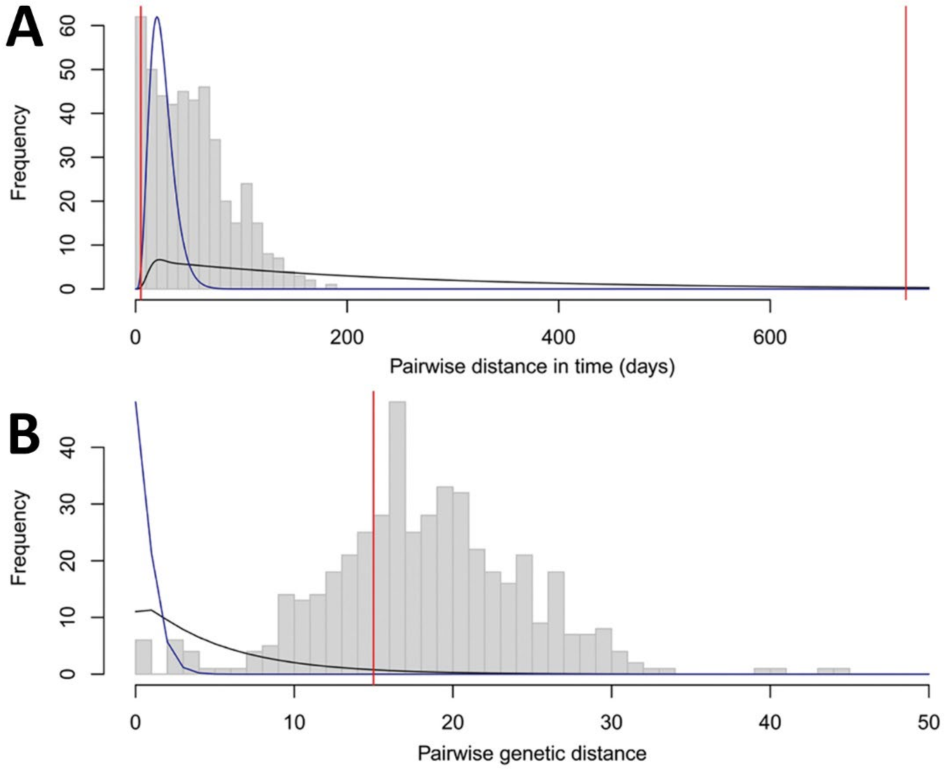

Appendix 1 Figure 3. Temporal and genetic distributions between paired sequenced dengue cases. The height of the bars indicates the number of case-pairs with specified temporal or genetic distance on the x-axis. The expected pairwise distance distributions for directly linked cases for (i.e. 100% reporting probability) are shown in blue. The expected pairwise distance distributions for linked cases when the reporting probability is 10% is shown in black. Pruning cutoffs (as described in Methods) are indicated by red vertical lines.
